# Supplementary material for: Limited gene flow and pronounced population genetic structure of Eastern Massasauga (Sistrurus catenatus) in a Midwestern prairie remnant
Source: PLoS One. 2022 Mar 24;17(3):e0265666. doi: 10.1371/journal.pone.0265666 (PMC8947261; doi:10.1371/journal.pone.0265666)
Supplement: S1 Table — Estimates were derived using Structure Selector [52] for 21 microsatellite markers across 327 Eastern Massasauga Rattlesnake individuals from Carlyle Lake, Illinois, USA. (DOCX) [file pone.0265666.s001.docx]

| K | Reps | Mean LnP(K) | Stdev LnP(K) | Ln'(K) | \|Ln''(K)\| | Delta K |
| --- | --- | --- | --- | --- | --- | --- |
| 1 | 10 | -21951.03 | 0.462 | NA | NA | NA |
| 2 | 10 | -20236.62 | 0.62147 | 1714.41 | 646.4 | 1040.11825 |
| 3 | 10 | -19168.61 | 0.89125 | 1068.01 | 827.37 | 928.32143 |
| 4 | 10 | -18927.97 | 27.85606 | 240.64 | 57.4 | 2.06059 |
| 5 | 10 | -18744.73 | 85.79981 | 183.24 | 41.37 | 0.48217 |
| 6 | 10 | -18602.86 | 78.30044 | 141.87 | 6.53 | 0.0834 |
| 7 | 10 | -18454.46 | 56.14058 | 148.4 | 22.62 | 0.40292 |
| 8 | 10 | -18328.68 | 76.14572 | 125.78 | 10.65 | 0.13986 |
| 9 | 10 | -18213.55 | 48.4722 | 115.13 | 56.67 | 1.16912 |
| 10 | 10 | -18155.09 | 88.20409 | 58.46 | NA | NA |

**S1 Table. The rate in change (ΔK) between successive K values.**

Estimates were derived using Structure Selector (Lu and Lui, 2018) for 21 microsatellite markers across 327 Eastern Massasauga Rattlesnake individuals from Carlyle Lake, Illinois, USA.
