# Supplementary material for: Limited gene flow and pronounced population genetic structure of Eastern Massasauga (Sistrurus catenatus) in a Midwestern prairie remnant
Source: PLoS One. 2022 Mar 24;17(3):e0265666. doi: 10.1371/journal.pone.0265666 (PMC8947261; doi:10.1371/journal.pone.0265666)
Supplement: S2 Table — Estimates were derived using Structure Selector [52] for 21 microsatellite markers across 115 Eastern Massasauga Rattlesnake individuals from Carlyle Lake, Illinois, USA. (DOCX) [file pone.0265666.s002.docx]

| K | Reps | Mean LnP(K) | Stdev LnP(K) | Ln'(K) | \|Ln''(K)\| | Delta K |
| --- | --- | --- | --- | --- | --- | --- |
| 1 | 5 | -7388.50 | 0.30000 | NA | NA | NA |
| 2 | 5 | -7137.12 | 2.69852 | 251.38 | 104.50 | 38.72496 |
| 3 | 5 | -6990.24 | 1.98947 | 146.88 | 23.30 | 11.71165 |
| 4 | 5 | -6866.66 | 6.99771 | 123.58 | 24.36 | 3.48114 |
| 5 | 5 | -6767.44 | 44.15538 | 99.22 | 6.82 | 0.15445 |
| 6 | 5 | -6661.40 | 36.12340 | 106.04 | NA | NA |

**S2 Table. The rate in change (ΔK) between successive K values for Eldon Hazlet State Park (EHSP).**

Estimates were derived using Structure Selector (Lu and Lui, 2018) for 21 microsatellite markers across 115 Eastern Massasauga Rattlesnake individuals from Carlyle Lake, Illinois, USA.
