# Supplementary figures and images for: Limited gene flow and pronounced population genetic structure of Eastern Massasauga (Sistrurus catenatus) in a Midwestern prairie remnant
Source: PLoS One. 2022 Mar 24;17(3):e0265666. doi: 10.1371/journal.pone.0265666 (PMC8947261; doi:10.1371/journal.pone.0265666)

# Delta K

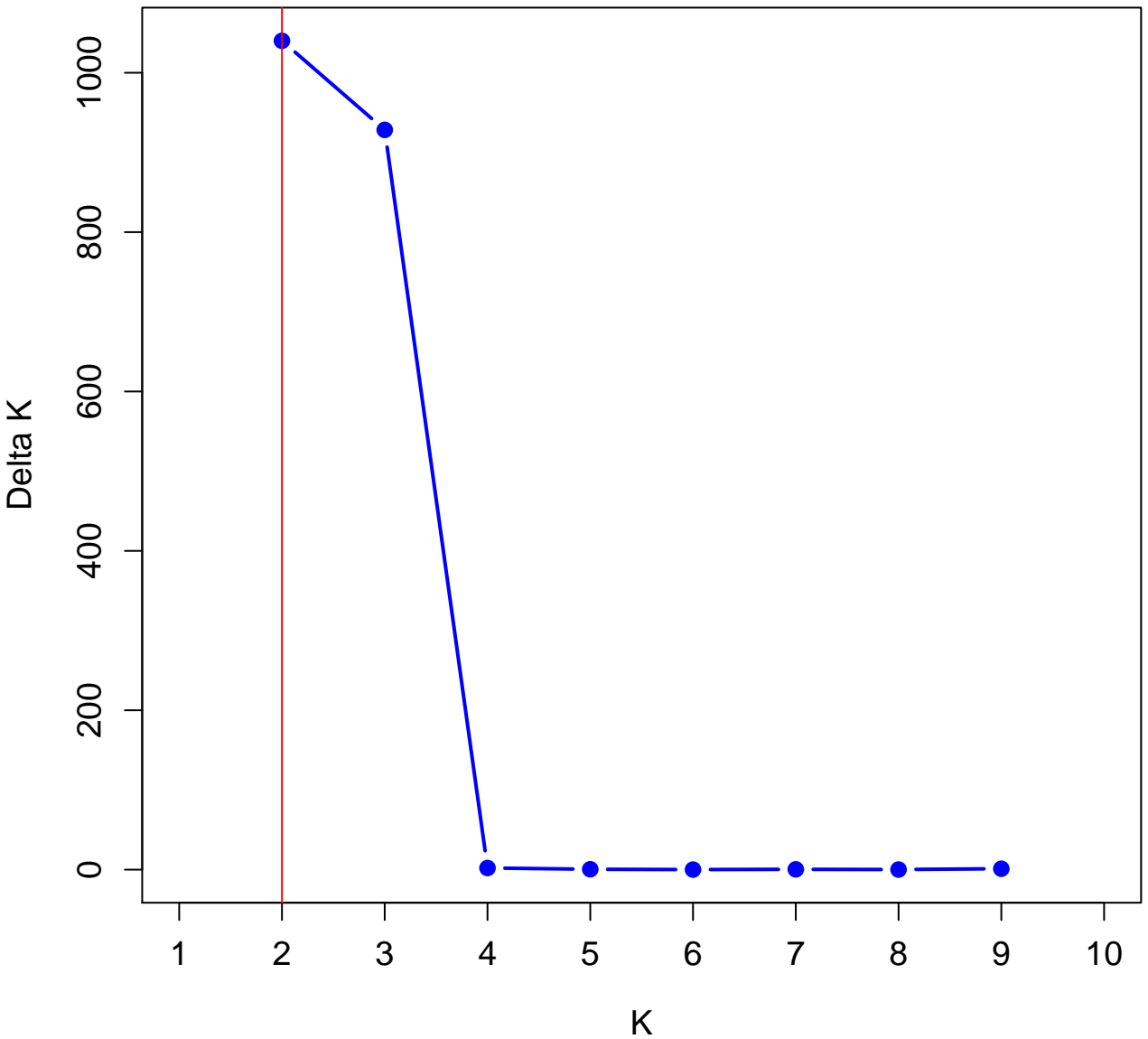

Supplement: S1 Fig — (PDF) [file pone.0265666.s003.pdf]

# Delta K

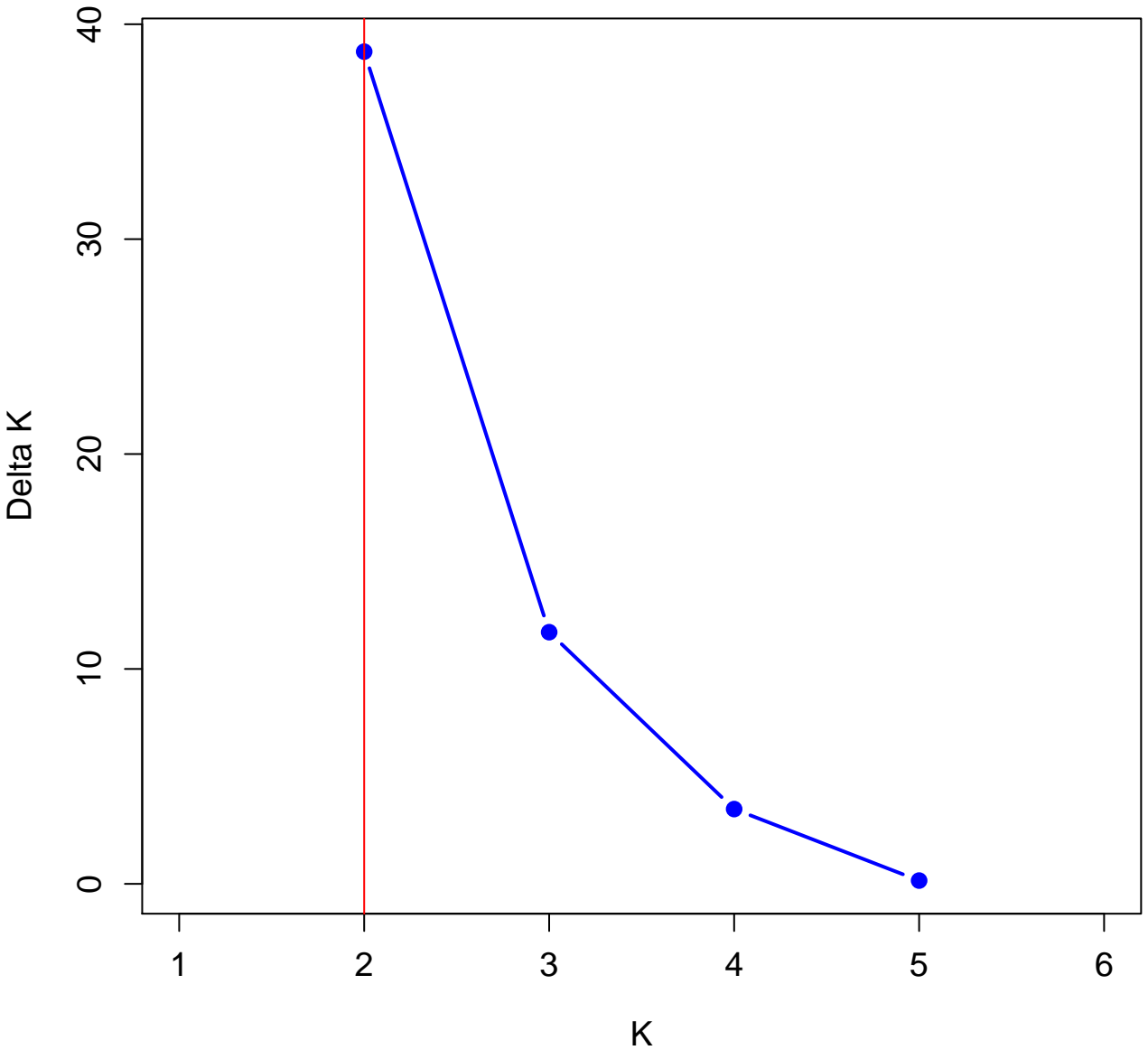

Supplement: S2 Fig — (PDF) [file pone.0265666.s004.pdf]
