# Supplementary material for: Limited gene flow and pronounced population genetic structure of Eastern Massasauga (Sistrurus catenatus) in a Midwestern prairie remnant
Source: PLoS One. 2022 Mar 24;17(3):e0265666. doi: 10.1371/journal.pone.0265666 (PMC8947261; doi:10.1371/journal.pone.0265666)
Supplement: S2 File — (PDF) [file pone.0265666.s006.pdf]

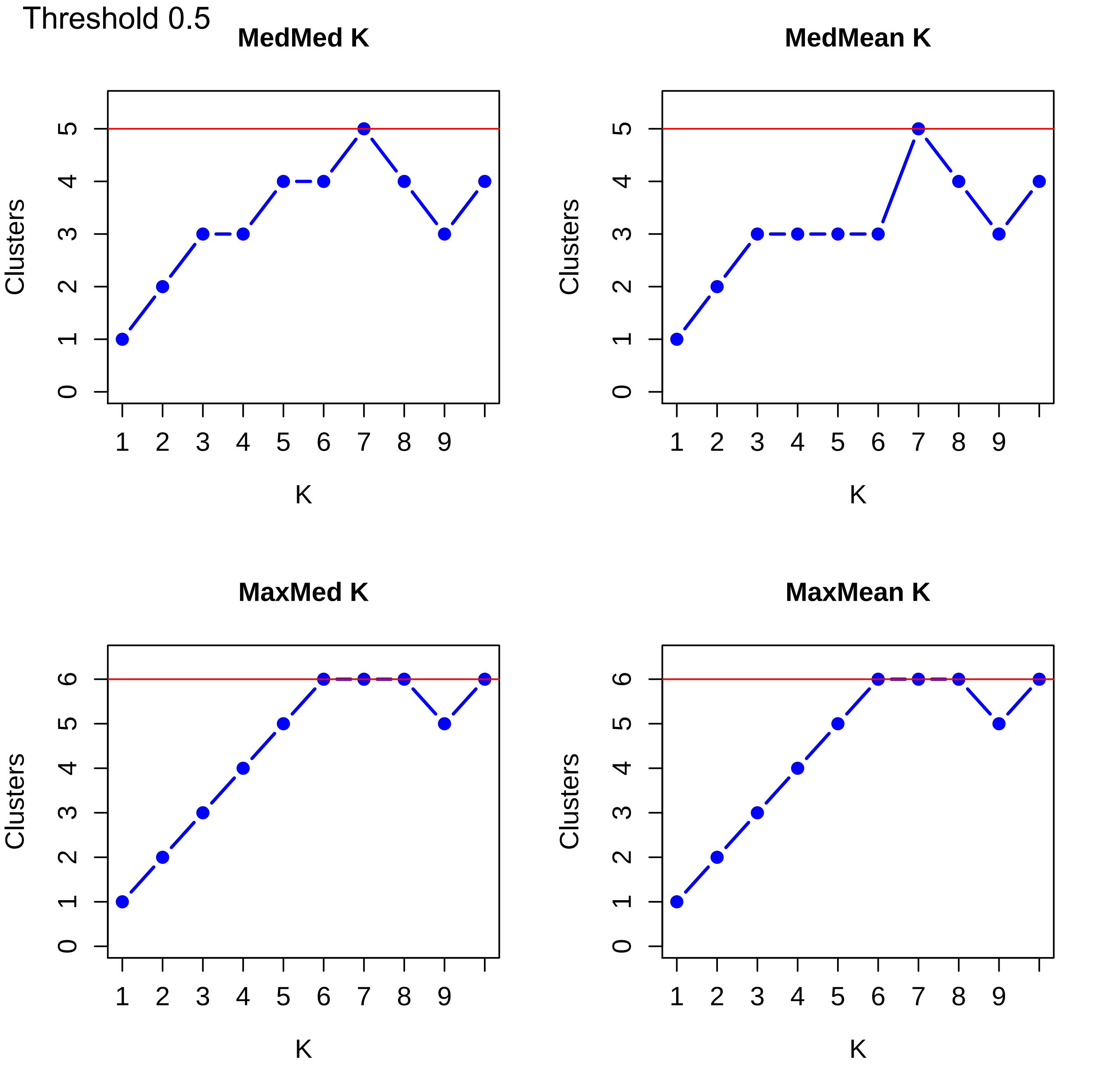
Threshold 0.5
MedMed K
MedMean K
MaxMed K
MaxMean K
Clusters
K
0
1
2
3
4
5
6
7
8
9

Threshold 0.6

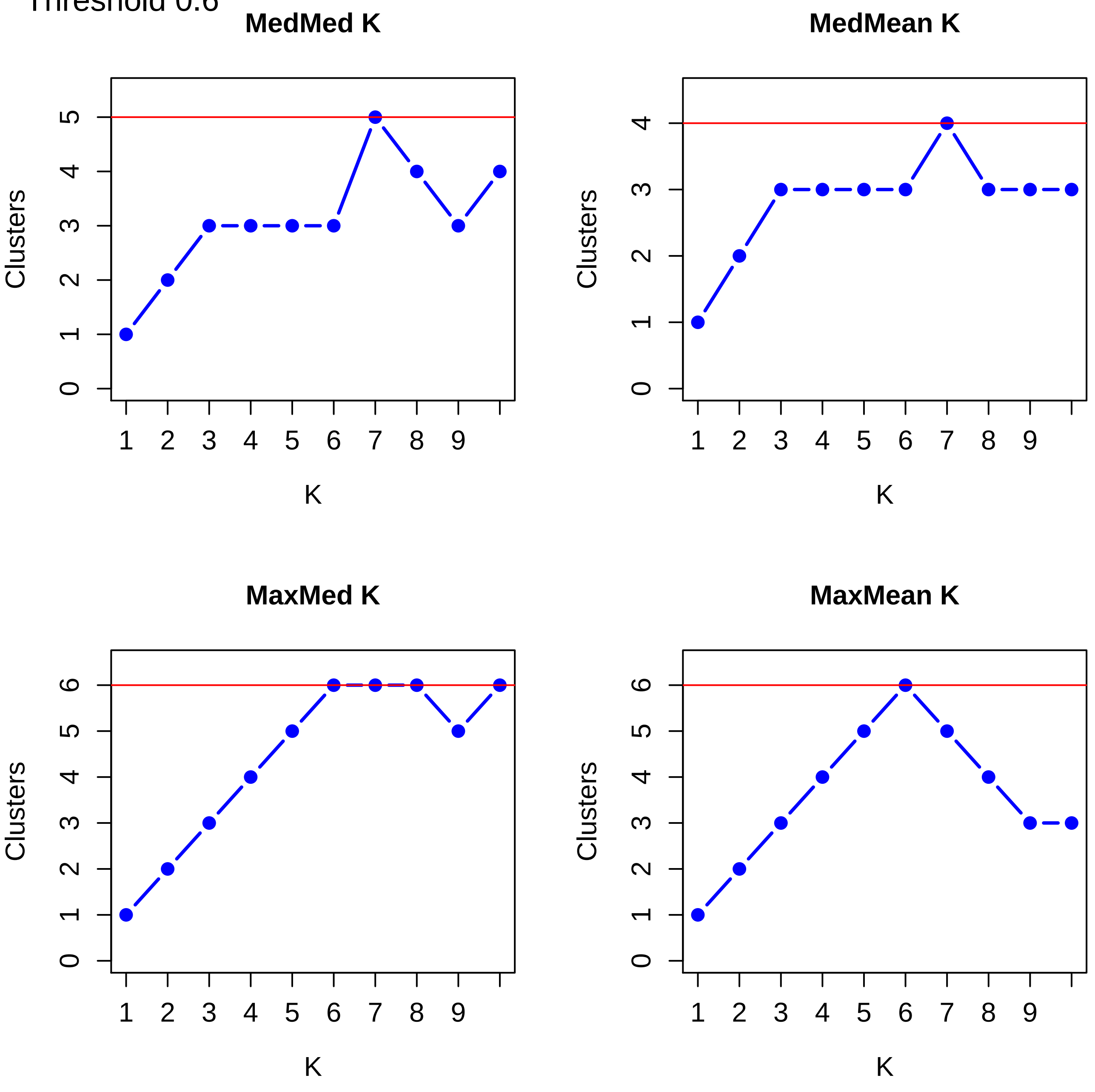

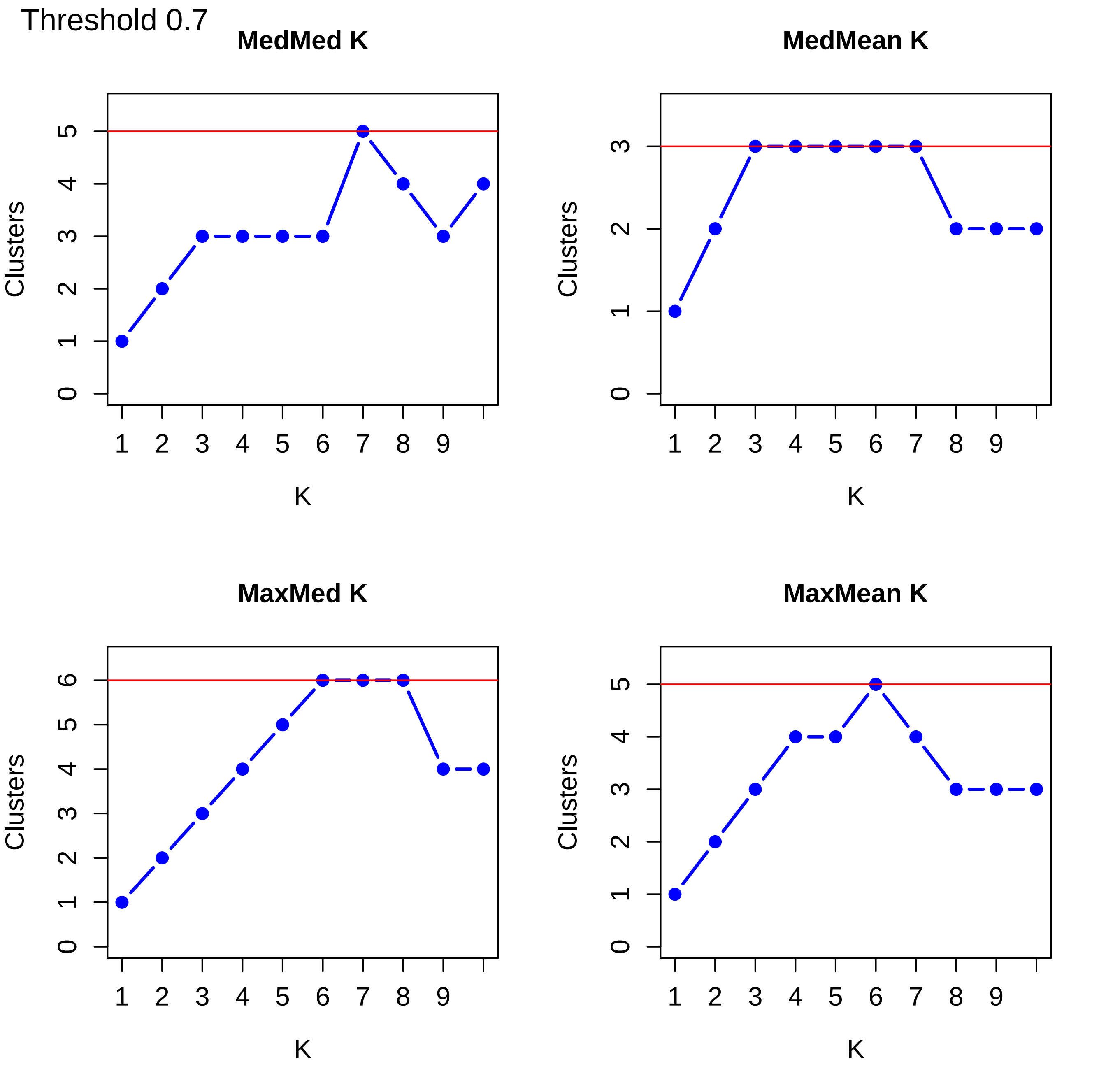

Threshold 0.7
MedMed K
MedMean K
MaxMed K
MaxMean K
Clusters
K

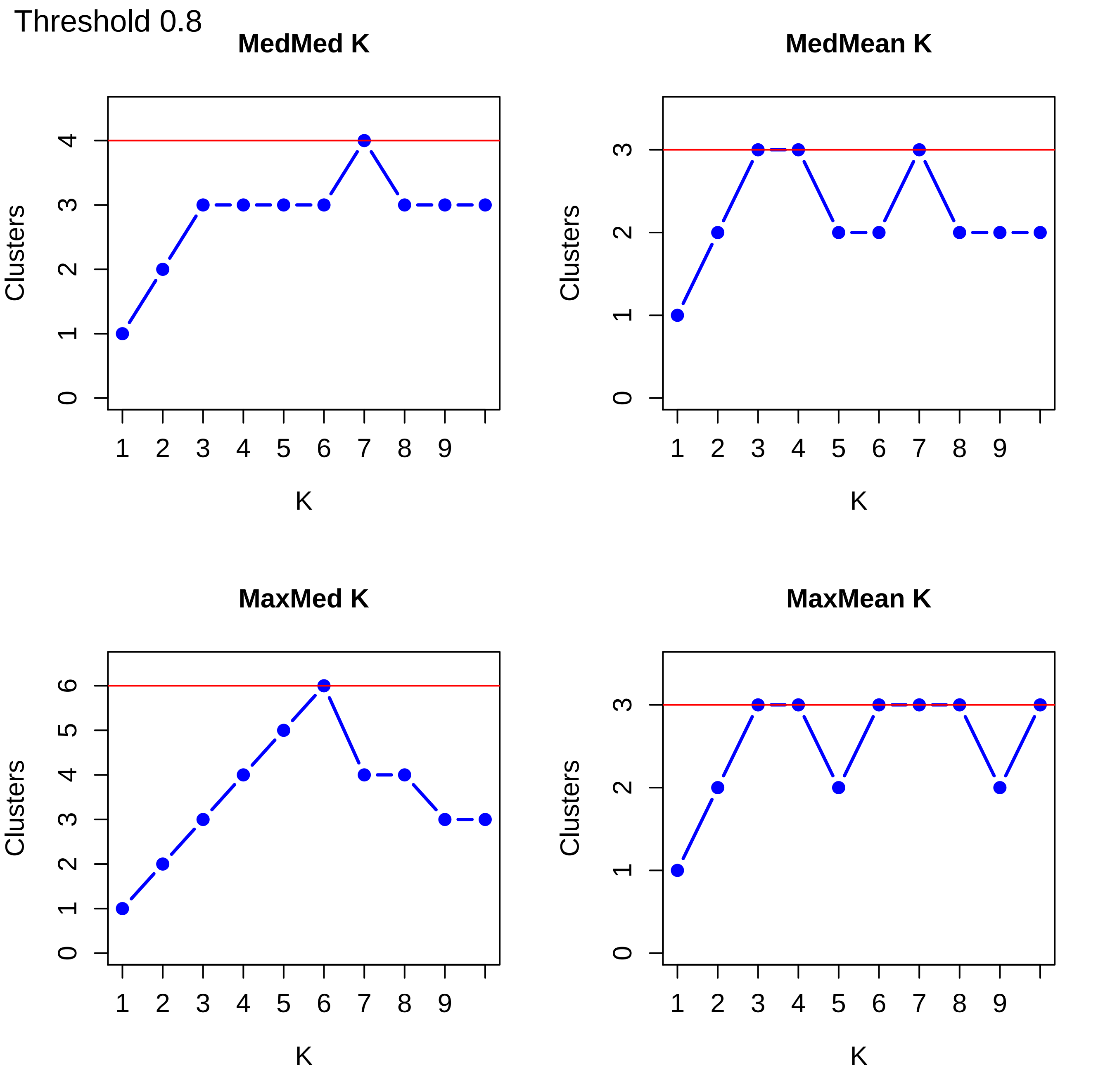
Threshold 0.8
MedMed K
MedMean K
MaxMed K
MaxMean K
Clusters
K
1 2 3 4 5 6 7 8 9
0 1 2 3 4
0 1 2 3
0 1 2 3 4 5 6
0 1 2 3
