# Supplementary material for: Limited gene flow and pronounced population genetic structure of Eastern Massasauga (Sistrurus catenatus) in a Midwestern prairie remnant
Source: PLoS One. 2022 Mar 24;17(3):e0265666. doi: 10.1371/journal.pone.0265666 (PMC8947261; doi:10.1371/journal.pone.0265666)

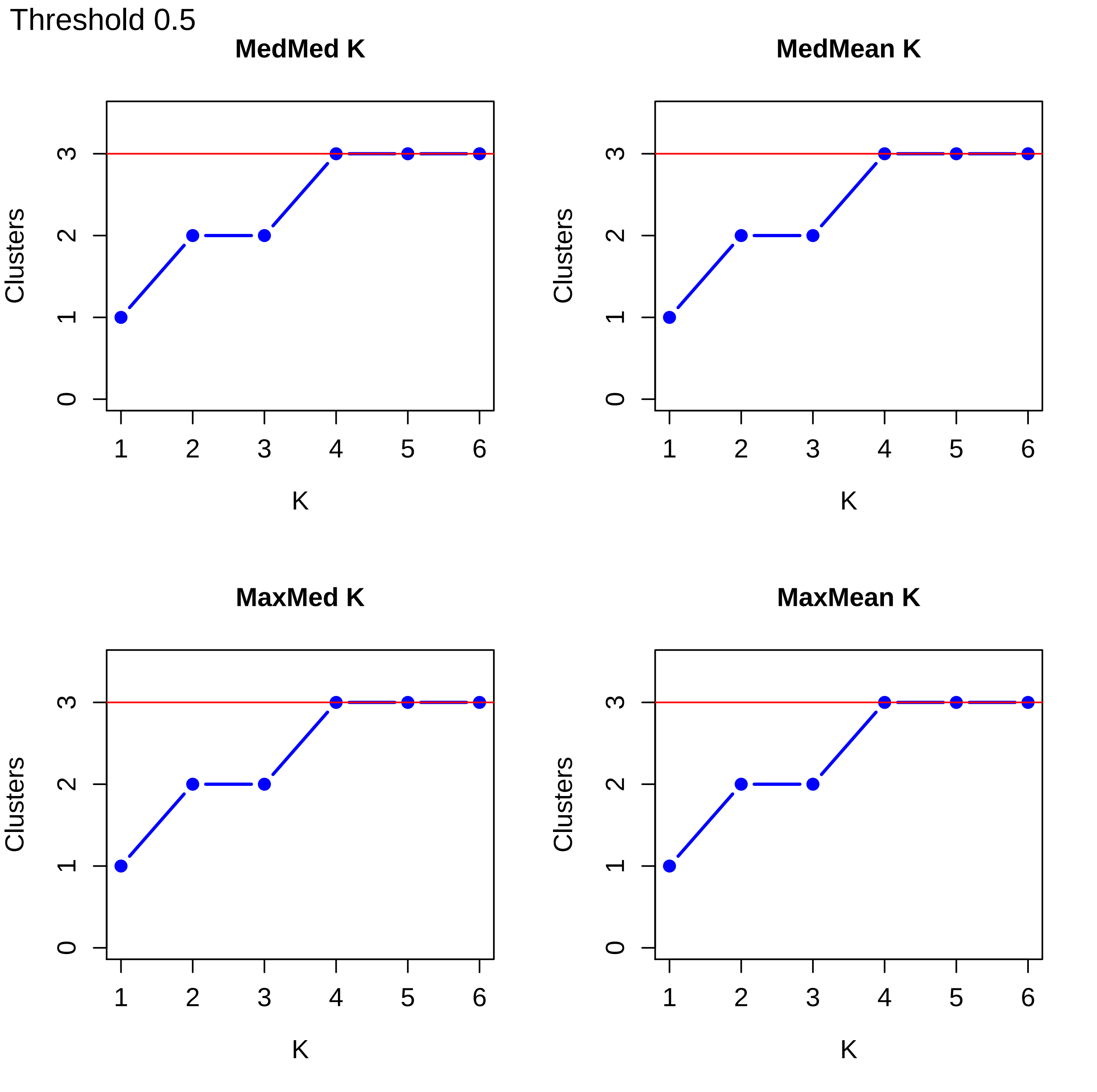
Threshold 0.5
MedMed K
MedMean K
MaxMed K
MaxMean K
Clusters
K
0
1
2
3
4
5
6

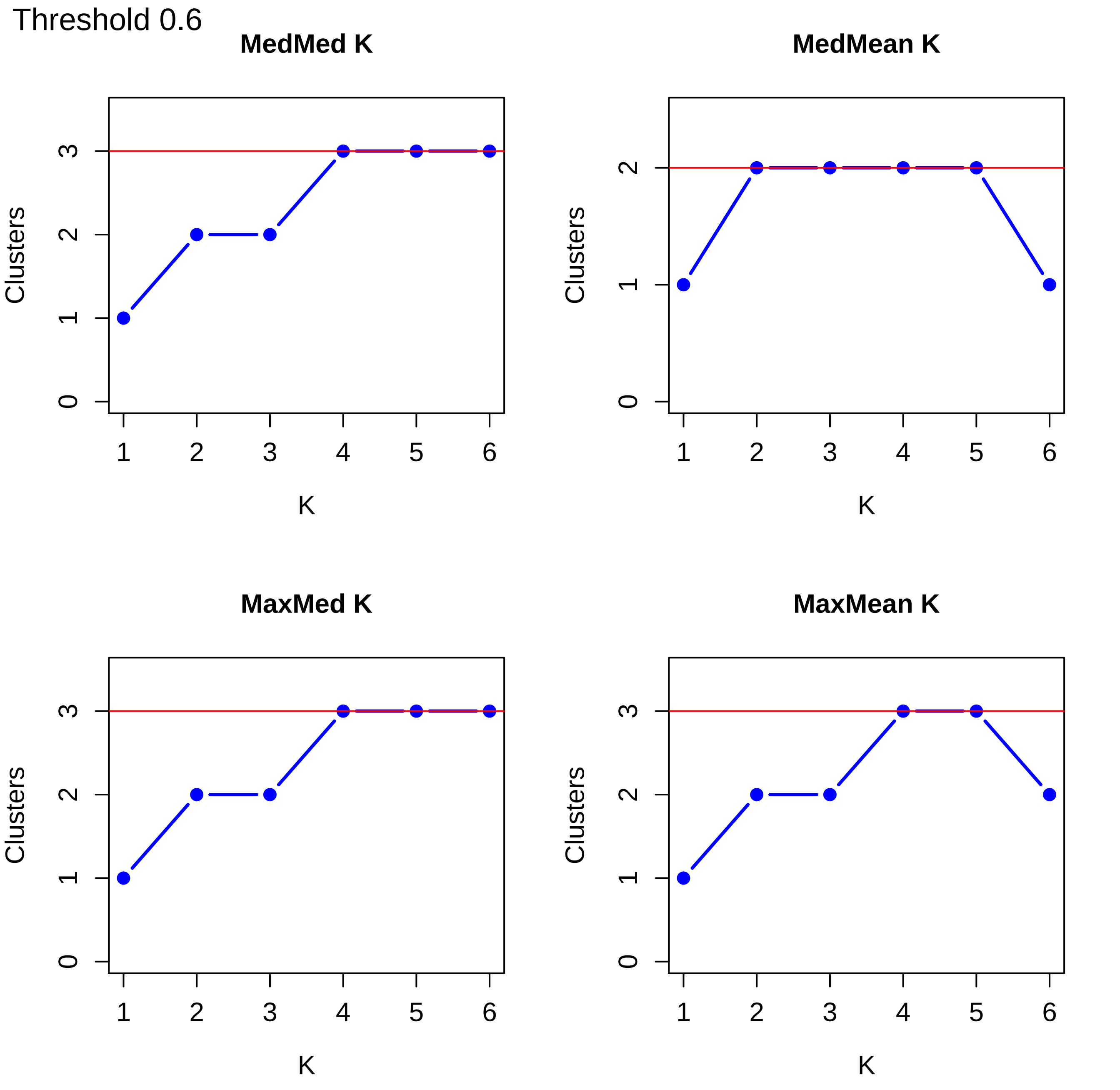
Threshold 0.6
MedMed K
MedMean K
MaxMed K
MaxMean K
Clusters
K
0
1
2
3
1
2
3
4
5
6

Threshold 0.7

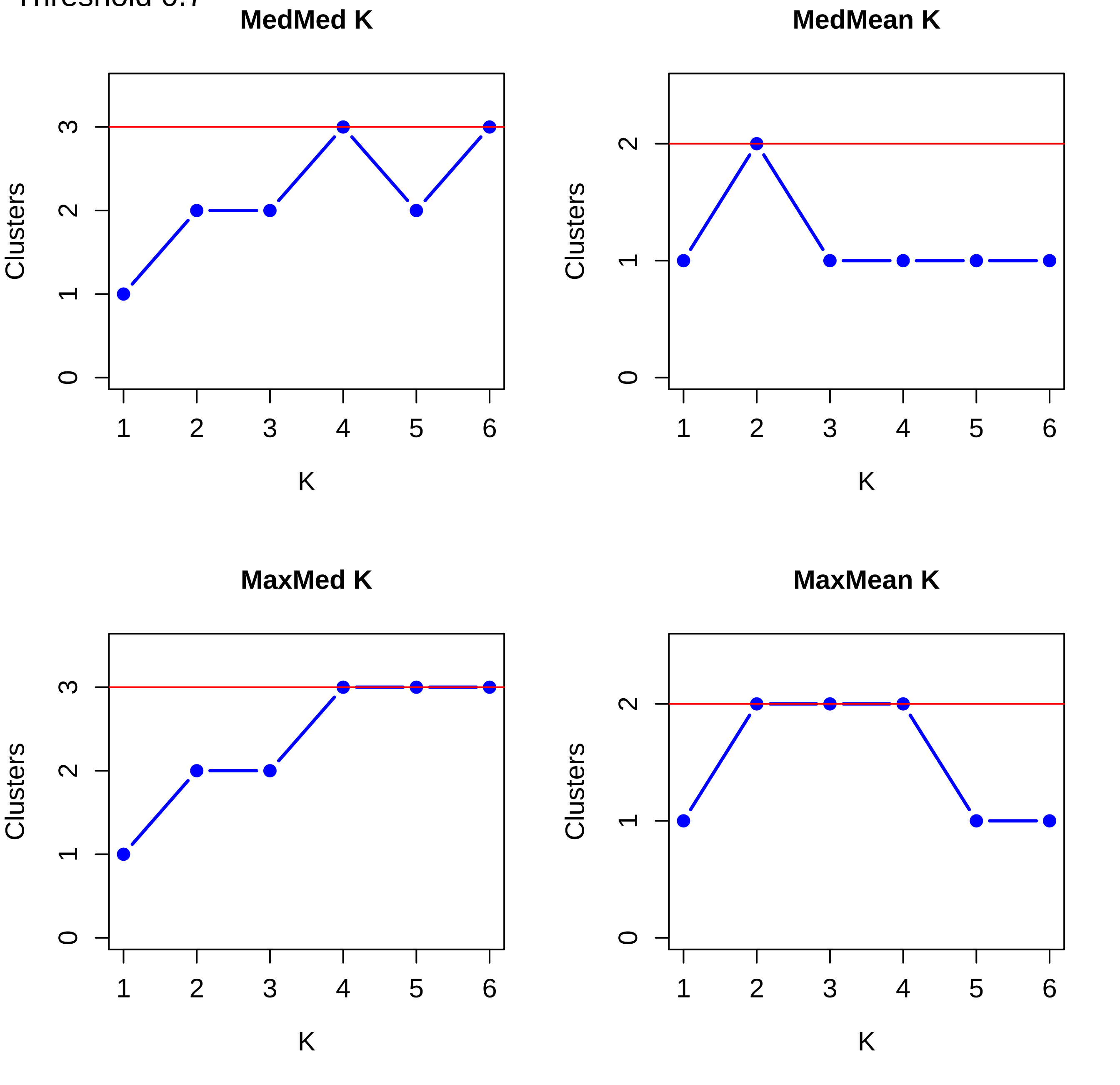

Threshold 0.8

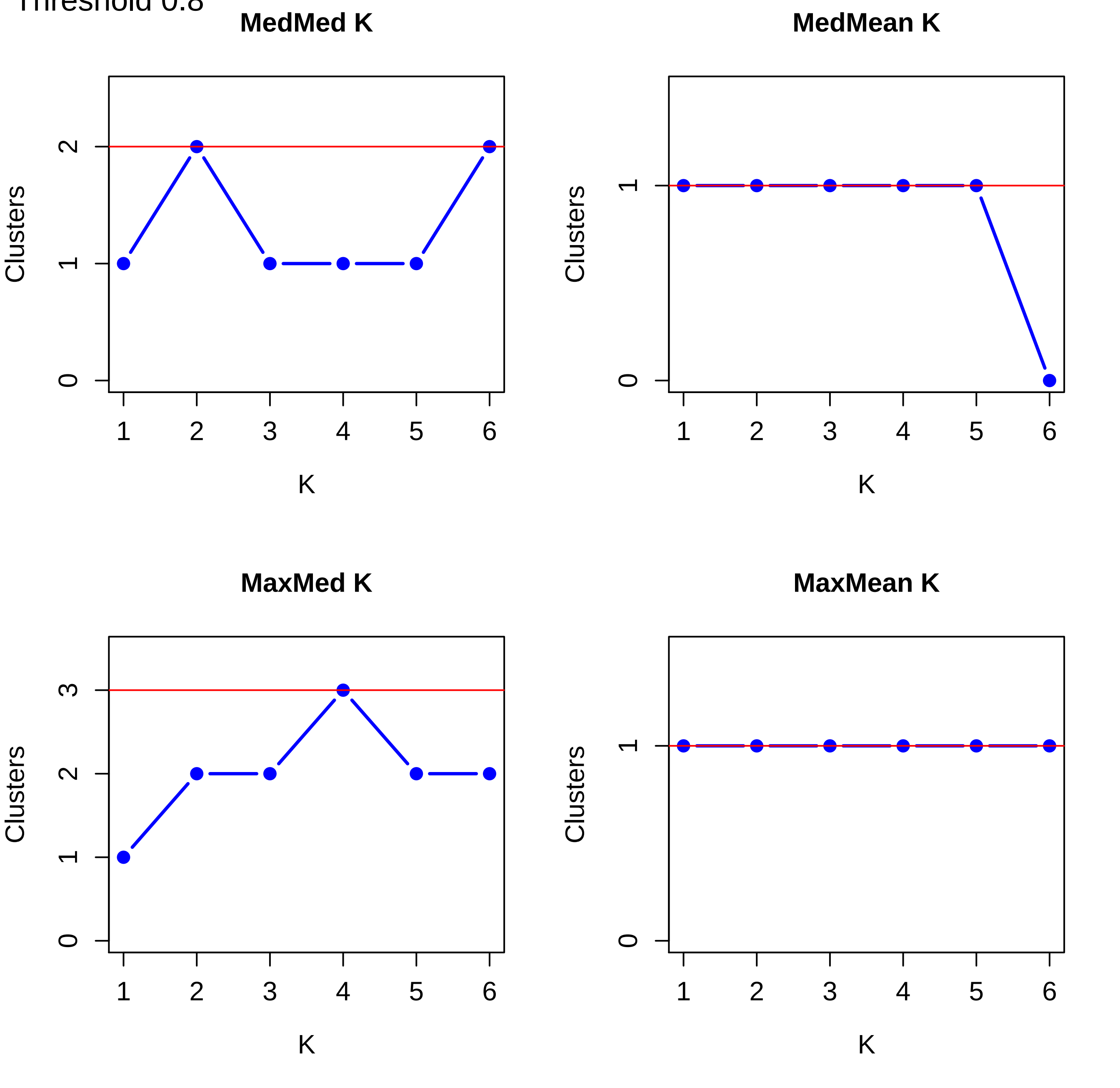

Supplement: S3 File — (PDF) [file pone.0265666.s007.pdf]
