# Supplementary material for: Limited gene flow and pronounced population genetic structure of Eastern Massasauga (Sistrurus catenatus) in a Midwestern prairie remnant
Source: PLoS One. 2022 Mar 24;17(3):e0265666. doi: 10.1371/journal.pone.0265666 (PMC8947261; doi:10.1371/journal.pone.0265666)
Supplement: Attachment — Submitted filename: Response to Reviewers.docx [file pone.0265666.s008.docx]

We have made the following required changes:

1. Updated Figure 1 to include a larger geographic context for the study site.
2. Expanded the discussion to provide the readers with more guidance about historical vs. contemporary fragmentation as well as the effects of fragmentation. Sections where this has been addressed include in the Discussion under the headings, Population structure and gene flow (starting at line 271) and Genetic diversity (starting at line 305). We did not incorporate and additional analysis (dataset simulation) as suggested by Reviewer 1 as we feel that we have addressed the concern to include a undisturbed population with changes to the text. For example, we discuss how it can be difficult to separate the effects of historical and contemporary landscape disturbance when natural features coexist with historic ones. Further, we acknowledge that recent genomic work (Ochoa and Gibbs, 2021) suggests that Eastern Massasauga has persisisted historically (10,000 ybp) at low Ne which could lead to the argument that we might expect strong population structure. However, we also point out that the same genomic study also detected recent population bottlenecks corresponding to anthropogenic fragmentation. This study also detected high levels population inbreeding that result from small population size and and a lack of natural selection against moderately deleterious alleles, which will exacerbate threats to long-term persistence.
3. We have provided more focused comments throughout the Discussion about the impacts of anthropogenic/contemporary disruptions (see #2 above). Additionally, under the Discussion heading: Implications for recovery of Eastern Massasauga (starting at line 329), we added the following sentence at the very beginning of the section: “Anthropogenic fragmentation can erode genetic diversity in vulnerable Eastern Massasauga populations as contemporary barriers to dispersal prevent gene flow, increase inbreeding depression, reduce adaptive variation, thereby accelerating populations into an extinction vortex (5,7,8).” We have also elaborated on management recommendations, citing previous studies that have made ecological-related management recommendations, and have added text to provide more explicit management recommendations with regard to genetic rescue (translocations and captive rearing).

We have addressed the following:

Reviewer #1:

Major comments –

- Re: including less anthropogenically disturbed areas or “expected” genetic structure. See comments above for required change #2.
- We have provided a supplemental excel files that includes genotypes, location and year of collection for each sample used in the analyses. For the most part, samples were collected throughout the duration of the study. In addition, we have shown that there little temporal change in genetic diversity over a 10-year period in samples analyzed from South Shore State Park (Baker et al. 2018). In short, we do not expect our observed genetic structure to correspond to temporal discordance. We have acknowledged this concern in the Results (lines 193-195).
- We have now included the unbiased estimator, G’’ST, in our pairwise comparison of genetic structure, but have also retained our estimates of FST to make results comparable to previously published studies. See lines 141-142 in the Methods, lines 177-178 in the Results as well as Tables 2 and 3.
- We have expanded the discussion to elaborate on why no significant inbreeding was found in each area and hibernaculum despite limited gene flow and small Ne. We cite the Ochoa and Gibbs 2021 study that did detect inbreeding (FROH) at SSSP and we acknowledge the potential limitations of using microsatellite markers to detect inbreeding when He is moderate and Ne has been historically small (See lines 315-325).

Minor comments –

- We have included a brief description of how hibernacula were designated. “The primary study areas are separated by distances of ~3-5 km and hibernacula (contiguous low-lying mesic grasslands with low canopy cover) within study areas are separated by unsuitable habitat and anthropogenic boundaries from a few kilometers to a few hundred meters.” Lines 123-125.
- We have updated Fig 1 to present a zoomed out map of the area but we did not add specific hiberacula locations to protect the populations from collection and persecution. Lines 125-126.
- We have removed isolation-by-distance text. Line 184 in the tracked changes version.

Reviewer #2

Minor Comments –

- Re: distribution of crayfish burrows: Hibernacula were determined by contiguous suitable habitat, low lying wet grasslands with low canopy cover. Therefore, hibernacula represent grassland patches across the landscape separated by various habitat (woodland, shrub-scrub, river, pond/lake etc.) and anthropogenic (roads, trails, rail lines, urbanization etc..) boundaries. Thus, hibernacula are separated by at least one type of barrier but permeability of movements across barriers differs (snakes will move through woodlands more readily than across roadways). Yes, even within a grassland habitat patch, crayfish burrow density differs depending on how mesic the microhabitat is. By default, areas with more crayfish burrows have more Massasaugas hibernating in them. We have provided a better description of how hibernacula were designated in the methods. (See lines 123-125).
- We greatly appreciated the suggestion to use the method of Puechmaille (2016) and Structure Selector (Li and Liu 2018) to identify the optimal value of K and have implemented this approach in our revised version of the manuscript. See lines 143-151 in the Methods, lines 181-193 in the Results as well as updated Fig 2-3 and the addition of two new tables (Table 5-6) that compares the values of K across stepwise coefficient membership thresholds. We have also included all Structure Selector output plots in our Supplementary files.
- Unfortunately, we do not have estimates of demographic parameters (e.g., survival, fecundity, etc.) for the three populations. These have been estimated for SSSP (see Sarah Baker’s dissertation; Baker 2016), but sufficient numbers of recaptures are lacking for EHSP or DMRA to calculate robust estimates.
- Line 424: We double-checked the title of the article and it is correct. “Detecting the number of clusters of individuals using the software STRUCTURE: A simulation study”
- Tables 1 and 3 have been updated. The number “1” was a simple formatting issue that has been corrected.

We have also made the following changes to address the journal requirements:

1. Updated the formatting for the main body and title page to meet PLOS ONE style requirements. Updates are highlight in the marked version of the manuscript.
2. Provided an excel file (S1) of the raw genotype data for all samples used in analyses. This file also includes collection year and hibernaculum abbreviation for each sample as requested by the reviewers.
3. Updated the legend for Figure 1 (Study site map) to include the following statement: All map data (road layer, municipal boundaries, streams, lakes, and protected lands) were publicly accessible and downloaded from the Illinois Geospatial Data Clearinghouse ([https://clearinghouse.isgs.illinois.edu/data](https://linkprotect.cudasvc.com/url?a=https%3a%2f%2fclearinghouse.isgs.illinois.edu%2fdata&c=E,1,Ow9METSRzqmFnMXjgecEQtb0ZnXr0KRwLvPfWbHWynsQ7sHea7v8d24M9UFR1kEJrD8lR4cak8xGapLR2172vjGtjSYMkRLSEu2tcera3gIE18FRGYrz6CvivEVY&typo=1)) and from I-View ([https://www.prairiestateconservation.org/pscc/iview/](https://linkprotect.cudasvc.com/url?a=https%3a%2f%2fwww.prairiestateconservation.org%2fpscc%2fiview%2f&c=E,1,djwe_MARPPb7MCzrQ0NxCeR6_Nc7LgZtzt7Mcfkt2GrishOvNGm3AB7pmgHwXdGeDWYviN1MzMOHNUZ3wFsFm1rrr_OXJCmERqepRx3I0UyN98k7FThm9BmIkE4,&typo=1)) and then projected in ARCMAP 10.8.0 (ESRI, Redlands, CA).
